# Horizontal transmission of the symbiotic bacterium *Asaia* sp. in the leafhopper *Scaphoideus titanus* Ball (Hemiptera: Cicadellidae)

**DOI:** 10.1186/1471-2180-12-S1-S4

**Published:** 2012-01-18

**Authors:** Elena Gonella, Elena Crotti, Aurora Rizzi, Mauro Mandrioli, Guido Favia, Daniele Daffonchio, Alberto Alma

**Affiliations:** 1 Dipartimento di Valorizzazione e Protezione delle Risorse Agroforestali (DIVAPRA), Università degli Studi di Torino, 10095 Grugliasco (TO), Italy; 2 Dipartimento di Scienze e Tecnologie Alimentari e Microbiologiche (DiSTAM), Università degli Studi di Milano, 20133 Milan, Italy; 3 Dipartimento di Biologia Animale (DBA), Università di Modena e Reggio Emilia, 41100 Modena, Italy; 4 Scuola di Bioscienze e Biotecnologie, Università degli Studi di Camerino, 62032 Camerino (MC), Italy

## Abstract

**Background:**

Bacteria of the genus *Asaia* have been recently recognized as secondary symbionts of different sugar-feeding insects, including the leafhopper *Scaphoideus titanus*, vector of Flavescence dorée phytoplasmas. *Asaia* has been shown to be localized in *S. titanus* gut, salivary glands and gonoducts and to be maternally transmitted to the progeny by an egg smearing mechanism. It is currently not known whether *Asaia* in *S. titanus* is transmitted by additional routes. We performed a study to evaluate if *Asaia* infection is capable of horizontal transmission *via* co-feeding and venereal routes.

**Results:**

A Gfp-tagged strain of *Asaia* was provided to *S. titanus* individuals to trace the transmission pathways of the symbiotic bacterium. Co-feeding trials showed a regular transfer of bacterial cells from donors to recipients, with a peak of frequency after 72 hours of exposure, and with concentrations of the administrated strain growing over time. Venereal transmission experiments were first carried out using infected males paired with uninfected females. In this case, female individuals acquired Gfp-labelled *Asaia*, with highest infection rates 72-96 hours after mating and with increasing abundance of the tagged symbiont over time. When crosses between infected females and uninfected males were conducted, the occurrence of “female to male” transmission was observed, even though the transfer occurred unevenly.

**Conclusions:**

The data presented demonstrate that the acetic acid bacterial symbiont *Asaia* is horizontally transmitted among *S*. *titanus* individuals both by co-feeding and venereal transmission, providing one of the few direct demonstrations of such a symbiotic transfer in Hemiptera. This study contributes to the understanding of the bacterial ecology in the insect host, and indicates that *Asaia* evolved multiple pathways for the colonization of *S*. *titanus* body.

## Background

*Asaia* is a genus of acetic acid bacteria belonging to the family Acetobacteriaceae [1,2], which resides in different environments, such as plants, flowers, herbs, fruits, and fermented foods and beverages. In recent years, bacteria of this genus have been observed infecting insects belonging to different orders, including Diptera, Hemiptera, Hymenoptera and Lepidoptera. Several of the species known to be stably associated with *Asaia* are important vectors of human interest (e.g. *Anopheles* and *Aedes* mosquitoes) or vectors of plant disease. *Scaphoideus titanus* Ball is in this category. *S. titanus* is involved in the diffusion of a plant disease, as it transmits “*Candidatus* Phytoplasma vitis”, the agent of Flavescence dorée (FD) of grapevine. Phytoplasmas are cell wall-less phloem-restricted bacteria of the phylum Mollicutes which induce serious diseases in plants and are often major causes of production losses for several crops. In the case of European viticulture the yield reduction caused by FD phytoplasma infections entails a very high economic damage [3].

A common trait of *Asaia*’s hosts is the fact they feed on sugar-based diets, suggesting this bacterium could have a role in nutrient metabolism [2]. Experiments with fluorescent *Asaia* strains supplied to the mosquitoes *Anopheles* spp. and *Aedes aegypti* Linnaeus, and the leafhopper *S. titanus* showed that this bacterium is able to colonize, re-colonize and cross-colonize the gut system, the gonads and the salivary glands [4,5]. The prevalence of *Asaia* in several insect host populations has been shown to be both stable and very high, suggesting it is not only an occasional commensal [4,6,7]. However the absence of phylogenetic congruency between *Asaia* isolates and their hosts indicates that these symbionts have been acquired by their hosts only recently, and can be transferred among different insect groups [2]. These features indicate that *Asaia*, along with other acetic acid bacteria colonizing different insects, can be considered as secondary symbiont [21] whose function in the hosts is not yet fully identified.

The ability of this bacterium to invade different organs of its insect host suggests that *Asaia* can be transmitted by a variety of transmission routes, both vertical and/or horizontal. Many symbiotic bacteria, like primary symbionts and several secondary symbionts, are vertically transmitted *via* the maternal route. Facultative symbionts may be also horizontally transferred, with feeding representing one of the main routes. For phloem feeding insects, transmission can occur when several individuals feed on the same plant [8-10], but transmission can also take place between host and parasitoid [11,12], or between parasitoids sharing the same host species [13,14]. In termites, horizontal transmission of gut bacteria has also been thought to occur *via* trophallaxis [16]. Another route of horizontal transmission is transfer during copulation, for example by the introduction of ejaculate components from male to female during copulation [15]. Moreover, experimental transinfection by means of hemolymph microinjections demonstrated the possibility of horizontal transfer *via* hemolymph sharing [17,18].

The vertical transmission of *Asaia* in *Anopheles stephensi* Liston, *Ae. aegypti* and *S. titanus* has been illustrated by Crotti *et al*. [4], who demonstrated the transmission of the symbiont *via* egg smearing, *i.e.* by contamination of the egg surface with bacterial cells by the mother, followed by the acquisition by the hatched offspring by consuming or probing the egg. In mosquitoes of the genus *Anopheles*, *Asaia* has been shown to infect through both per-oral [6,19] and venereal routes, from male to female, followed in each case by vertical spread from the mother to the offspring [5,20]. These transmission routes are in agreement with both the incongruent evolutionary history of *Asaia* and its host species, and with the high frequency of infections with multiple *Asaia* strains in mosquitoes [21]. However, very little is known about the rate and mechanisms of horizontal transfer of *Asaia* in hemipterans like *S. titanus*. Horizontal transfer in this species has been only indirectly demonstrated by the capability of *Asaia* to be established in leafhopper individuals fed with bacterial cells and by the ability to colonize insect salivary glands [2].

The exploitation of symbiotic microorganisms of insect vectors is recently emerging as a strategy to limit the diffusion of arthropod-borne diseases through the development of “symbiotic control” strategies [22]. This approach could represent a promising alternative to current FD control methods, which are limited to the use of chemical insecticides and to the removal of infected plants. To set up a symbiotic control strategy, a microbial symbiont that meets the requirements needed for a control agent must be firstly identified. Such requirements include stable association with the vector, dominance within its microbial community, co-localization with the pathogen, predisposition to *in vitro* manipulation, and, last but not least, an efficient spread system within insect populations [23]. *Asaia* and other acetic acid bacteria have such features in relation to dipteran mosquitoes, so they have been indicated as potential agents for natural or paratransgenic symbiotic control [4,6,24]. However, the capacity of *Asaia* to be transmitted horizontally among *S. titanus* has not been yet investigated.

The objective of this work was to evaluate whether *Asaia* is horizontally transmitted among *S. titanus* individuals by the oral and the venereal transmission routes. This could contribute to the evaluation of the ecology of this acetic acid bacterium in leafhopper populations.

## Results and discussion

### Donor insects

**Figure 1:**
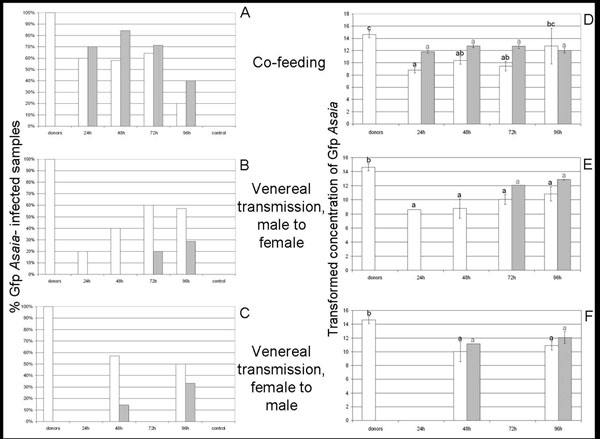
**Gfp-Asaia infection rates and density within infected samples.** White columns represent *S. titanus* individuals, and grey columns represent diets. The “donors” columns refer to the average values of donor insects in all of the trials. “24h”, “48h”, “72h”, and “96h” indicate the time of exposure to co-feeding or the time of incubation after mating with infected individuals. The “control” columns represent the values obtained from insects fed on sterile sugar diets, as well as those obtained from individuals co-housed with Gfp *Asaia*-infected specimens of the same sex. A-C) Percentage of insects and diets colonized by Gfp-tagged *Asaia*. D-F) Transformed (10 + log) number of *gfp* gene copies per positive sample. Bars represent the standard error of transformed data. Different letters (black for insect and grey for diet samples) indicate significantly different values (ANOVA, P<0.05).

**Figure 2:**
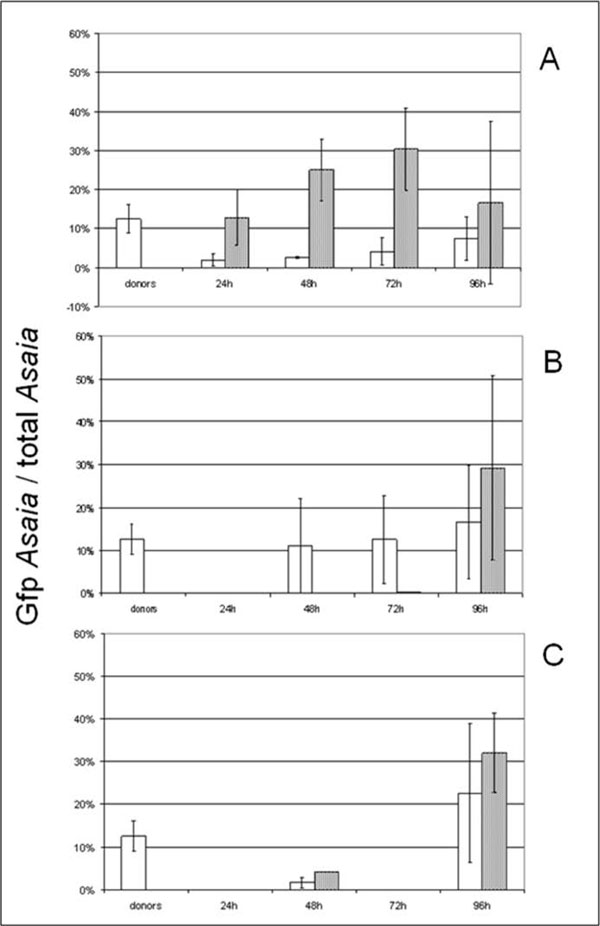
**Relative abundance of **G**fp-Asaia within the whole Asaia populations.** The relative abundance of the tagged strain in total *Asaia* community is calculated by the ratio between the number of *gfp* gene copies per sample and the number of *Asaia* cells (which is *Asaia* 16S rRNA gene copies divided by four, assuming that four rRNA gene copies per cell are present in *Asaia*, as reported in Crotti et al. [4]) per sample. In each graph white columns represent *S. titanus* individuals, and grey columns represent diets. The “donors” columns refer to average values of donor insects in all trials. “24h”, “48h”, “72h”, and “96h” indicate the time of exposure to co-feeding or the time of incubation after mating with infected individuals. The Gfp-tagged *Asaia* to total *Asaia* ratio is indicated in insects and diets submitted to co-feeding trials (A), and to venereal transmission experiments, from male to female (B) and from female to male (C), respectively. The bars on each column represent the standard error.

**Figure 3:**
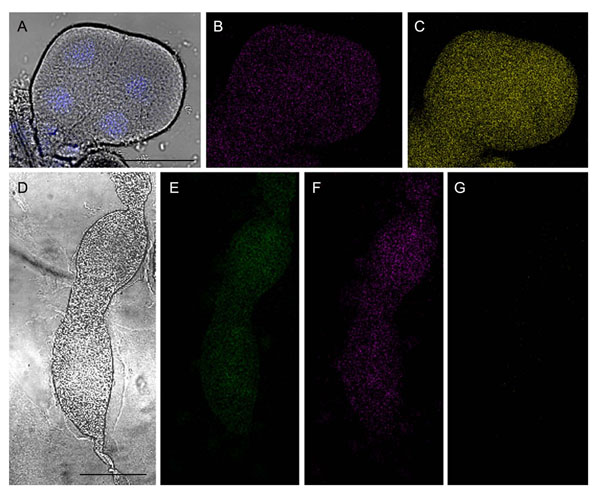
**Positive and negative controls for FISH experiments targeting the gfp gene.** The presence and distribution of Gfp-tagged *Asaia* in tissues of donor insects (positive controls) and of individuals submitted to transmission trials in absence of the tagged strain (negative controls) have been evaluated by FISH with the FITC-labelled Eu338 eubacterial probe (green), the Cy3-labelled *Asaia*-specific probes (magenta) and the Cy5.5-labeled probes specific for the *gfp* gene (yellow). A) Superposition of a CLSM image after staining with DAPI over the interferential contrast microscopy picture of a salivary gland lobe of an individual used as donor during co-feeding trials (bar = 50 µm). B,C) CLSM images after hybridization with the Cy3-tagged probes targeting the whole *Asaia* population (B), or with the Cy5.5-marked probes specific for the Gfp strain (C). In D-G) an ovariole of a female mated with a male which was not previously fed with the Gfp-tagged *Asaia* is shown. D) Interferential contrast micrograph showing the ovariole (bar = 150 µm). E-G) CLSM images of FISH with the FITC-labeled eubacterial probe (E), the Cy3-tagged probes targeting the whole *Asaia* population (F), and the Cy5.5-marked probes specific for the *gfp* gene (G). While the occurrence of bacteria (and *Asaia* in particular) is shown, no hybridization signal was observed with the *gfp* gene-specific probes.

Insects destined to test transmission of infection (‘donors’) were infected with a marked strain of *Asaia*. To this end, donors were fed with diets added of Gfp-tagged *Asaia* for 48 hours and then allowed to release the symbiont for 48 hours in diets supplemented with kanamycin. Afterwards the diets, in which Gfp-tagged *Asaia* was released, were exposed to recipient individuals for 24, 48, 72 and 96 hours, respectively. At the same time, the 98 individuals used as donor specimens were collected to be tested in q-PCR. All of them were positive for the *gfp* gene, with an average titre of 1.1 × 10^6 ^*gfp* gene copies / pg of insect 18S rRNA gene (Figure 1, Table 1). Furthermore, Gfp *Asaia* represented 12.5% of total *Asaia* population residing in the insect body (Figure 2), indicating that the newly-introduced symbionts colonized the insect body together with wild type *Asaia* that was already present in the individual. The proportion of the Gfp strain and of total *Asaia* in the whole bacterial community of donor individuals were 0.7% and 5.8%, respectively (Table 2). The *Asaia* to bacteria ratio (ABR) was similar to the value previously reported (4.9%) for populations of the symbiont in field-collected *S. titanus *[2]; the higher value found in this study could be attributed to the additional uptake of Gpf-tagged *Asaia* cells from the diets supplementing those naturally occurring in the insect. A further confirmation of colonization of the insect body by the Gfp-tagged *Asaia* was obtained by FISH experiments, which highlighted the acquisition by the insect of the tagged strain in different organs, including salivary glands (Figure 3 A-C). The colonization of salivary glands indicates that *Asaia* can be released into the feeding medium, potentially allowing bacterial transfer to other individuals.

**Table 1:** Gfp Asaia concentration in S. titanus individuals and in diets.

		insect		diet	
		average titre	standard deviation	average titre	standard deviation
	**donors**	1.1 × 10^6^	2.09 × 10^6^	-	-

Co-feeding	**24h**	4.75×10 ^-1^	8.77 × 10^-1^	1.84 × 10^2^	3.16 × 10^2^
	**48h**	2.14 × 10^2^	5.26 × 10^2^	3.03 × 10^3^	5.74 × 10^3^
	**72h**	2.67 × 10^3^	8.01 × 10^3^	2.22 × 10^3^	3.25 × 10^3^
	**96h**	2.32 × 10^5^	3.28 × 10^5^	3.85 × 10^3^	6.63 × 10^2^
	**control**	0	0	0	0

venereal transfer (male to female)	**24h**	3.96 × 10^-2^	-	0	0
	**48h**	6.73 × 10^-1^	9.48 × 10^-1^	0	0
	**72h**	8.06 × 10^0^	1.32 × 10^1^	1.14× 10^2^	-
	**96h**	8.96 × 10^2^	1.79 × 10^3^	7.27 × 10^2^	4.57 × 10^1^
	**control**	0	0	0.	0

venereal transfer (female to male)	**24h**	0	0	0	0
	**48h**	2.54 ×+02	4.42 × 10^2^	1.47 10^1^	-
	**72h**	0	0	0	0
	**96h**	2.53 ×+01	2.41 × 10^1^	4.13 × 10^2^	5.61 × 10^2^
	**control**	0	0	0	0
Co-housing	**24h**	0	0	0	0
	**48h**	0	0	0	0
	**72h**	0	0	0	0
	**96h**	0	0	0	0

The concentration of Gfp *Asaia* in insect and diet samples as indicated by the number of *gfp* gene copies per positive sample. In case of insect samples, the *gfp* copy number was calculated per pg of insect 18Sr RNA gene, while for diets it was calculated per ng of total DNA. Average values and standard deviation are shown for each trial. Standard deviation is missing when the number of positive samples was <2.

**Table 2:** Relative abundance of Gfp-tagged Asaia and Asaia sp. within the bacterial community of samples.

	GfpABR					ABR				
**Sample and transmission type**	**Average (SD)**	**24h**	**48h**	**72h**	**96h**	**Average (SD)**	**24h**	**48h**	**72h**	**96h**

Insect – Donors	0.00724 (0.03573)	-	-	-	-	0.05783	-	-	-	-
Insect –Co-feeding	0.00145 (0.00166)	0.0000004	0.00212	0.00349	0.00019	0.04239 (0.04745)	0.00002	0.08202	0.08490	0.00263
Insect –Venereal transfer, ♂ to ♀	0.00105 (0.00179)	0.0000003	0.00372	0.00004	0.00043	0.02277 (0.02602)	0.05436	0.03381	0.00032	0.00258
Insect –Venereal transfer, ♀ to ♂	0.00137 (0.00025)	-	0.00119	-	0.00155	0.04265 (0.05056)	-	0.07840	-	0.00690
Diet –Co-feeding	0.06143 (0.04979)	0.12291	0.02367	0.08079	0.01833	0.35694 (0.40712)	0.95646	0.09473	0.26633	0.11026
Diet –Venereal transfer, ♂ to ♀	0.00070 (0.00045)			0.00038	0.00102	0.09653 (0.13157)	-	-	0.18957	0.00350
Diet –Venereal transfer, ♀ to ♂	0.00490 (0.00501)	-	0.00135	-	0.00844	0.02983 (0.00491)	-	0.03330	-	0.02636

GfpABR (Gfp-tagged *Asaia* to Bacteria ratio) calculated as the ratio between the *gfp* copy number and the 16S rRNA gene copy number of the total bacterial community of the samples. ABR (*Asaia* to Bacteria ratio) calculated as the ratio between the number of *Asaia* cells and the total bacteria 16S rRNA gene copy number. In case of insect samples, all of the final copy numbers were calculated per pg of insect 18Sr RNA gene. Values in the Average column represent the average results of each group of trials for insect and diet samples; standard deviation is indicated in parenthesis.

### Co-feeding experiments

Donor individuals previously exposed to gfp *Asaia* were allowed to feed on artificial diets, and ‘recipient’ individuals then exposed to this diet. There was a high frequency of transfer of *Asaia* to both the food source and to *S. titanus* during feeding, as indicated in Figure 1A. The occurrence of *gfp* gene-positive signals in sugar diets previously exposed to donor insects confirms the earlier indications of a release of *Asaia* by *S. titanus* during feeding events [4]. The proportion of diets that assayed positive for Asaia showed a trend characterized by a peak corresponding to 48 hours post exposure to the donor (16 out of 19 positive samples; while 7 out of 10 samples were positive after 24 hours), followed by a decrease starting from the 72 hours acquisition (10 out of 14 positive samples; 4 out of 10 after 96 hours). The average concentration of the marked strain, calculated by the number of *gfp* gene copies per ng of DNA of the diet sample, increased up to 48 hours after the end of the inoculation (3 × 10^3 ^*gfp* gene copies / ng DNA) and then started decreasing reaching a value of 3.9 × 10^2 ^*gfp* gene copies / ng DNA after 96 hours acquisition (Table 1). The proportion of the Gfp strain within the total *Asaia* population followed a similar trend, increasing up to 30% at 72 hours, and decreasing after 96 hours (Figure 2A). This decline could be attributed to the occurrence of other bacteria that can compete with *Asaia* for the nutrient sources. Beside the highly frequent release of both Gfp- and wild type *Asaia* into the diet, other bacteria were inoculated into the feeding medium by *S. titanus*, as the GfpABR with ABR of 6% and 36% respectively (Table 2). Other bacteria associated with the leafhopper could also be transmitted during feeding events, including the phytoplasma and possibly the endosymbiont “*Candidatus* Cardinium hertigii”, observed to reside in *S. titanus* salivary glands [25]. However, even if Gfp *Asaia* concentration may be further reduced over time, the decline in concentration observed during the experiment was not significant (df= 36; F= 0.396; P= 0.879) (Figure 1D).

The quantitative PCR analysis performed on the DNA of recipient *S. titanus* individuals showed that when *Asaia* is inoculated into the sugar diet, it can be ingested by the insect and multiply in its body. Even though not all of the positive diets led to the development of an infected recipient insect, indicating that the acquisition process may fail, successful transmission was common (Figure 1A). The rate at which recipient individuals became infected remained stable around 60% at an acquisition time of 24 hours to 72 hours (6 out of 10 positive individuals after 24 hours; 11 out of 19 after 48 hours; 9 out of 14 after 72 hours). The rate declined after 96 hours of acquisition (2 out of 10), which is in accord with the decrease of Gfp-tagged *Asaia* in infected diets observed above. Despite the reduced number of stable long-term colonization events, Gfp-labelled *Asaia*, represented an average of 0.1% of the bacterial community in infected insects (Table 2),and showed high concentrations when insects fed for a longer period. In fact, the average titre of *Gfp*-tagged *Asaia* increased linearly over time passing from 4.8 × 10^-1^ copies of *gfp* genes per pg of insect 18S rRNA gene at 24 hours to 2.3 × 10^5^ copies of *gfp* genes per pg of insect 18S rRNA at 96 hours (Table 1), suggesting that *Asaia* succeeded in establishing within the host’s body. However, despite the continuous increase of Gfp *Asaia* concentration, the concentration values were significantly lower than that of donor individuals for co-feeding periods up to 72 hours (df=37; F=12.249; P<0.05). Only after a 96-hour co-feeding was a value not significantly different to that of donor individuals reached (Figure 1D). The ratio of the *Gfp* strain and total *Asaia* also followed a constantly rising trend, although even after 96 hours of acquisition the ratio was still much lower than that of donor individuals (Figure 2A). The increase of the Gfp/*Asaia* ratio suggests that the modified symbiont is able to compete with the naturally occurring *Asaia* within the insect body during the host’s colonization, without upsetting its population. In fact, the average percentage of total *Asaia* in the whole bacterial community of individuals submitted to co-feeding trials (4%) did not diverge from the normally observed ABR (4.9%) [4] (Table 2). In agreement with the co-infection of multiple *Asaia* strains within the same host that has been demonstrated for mosquitoes [21], further long term acquisition experiments could examine whether the two strains may co-exists for longer time periods in the same tissues after a horizontal transmission event.

**Figure 4:**
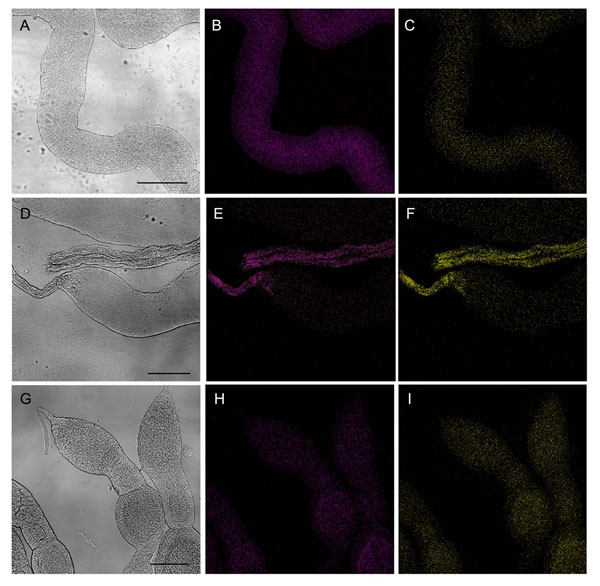
**Localization of horizontally-transmitted Gfp *Asaia* in organs of *S. titanus* individuals.** Images of insect tissues after hybridization with the Cy3-labeled *Asaia*-specific probes (magenta) and the Cy5.5-labeled probes specific for the *gfp* gene (yellow) showing the distribution of the symbiont within the gut, the ovaries and testes of specimens after acquisition of the tagged bacterium *via* co-feeding or venereal transmission. A-C) Midgut portion of an individual after 48-hour acquisition during the co-feeding trial, observed by interferential contrast microscopy (A) and CLSM after hybridization with the Cy3-tagged probes targeting the whole *Asaia* population (B), or with the Cy5.5-marked probes specific for the *gfp* gene(C). D-F) Testis portion of an individual after co-feeding trial observed by interferential contrast microscopy (D), and by CLSM after hybridization with the Cy3-tagged probes targeting the whole *Asaia* population (E) and the Cy5.5-marked probes specific for the *gfp* gene (F). In G-I) ovaries of a *S. titanus* individual after the acquisition in venereal transmission experiments are shown. G) Interferential contrast micrograph showing a group of ovarioles. H, I) CLSM images of FISH with the Cy3-tagged probes targeting the whole *Asaia* population (H) and the Cy5.5-marked probes specific for the *gfp* gene (I). Bars = 150 µm.

Once the ability of *Asaia* to be horizontally transmitted by sharing food source was verified, the details of colonization patterns within the insect body were examined by whole mount FISH experiments on organs dissected from *S. titanus* individuals after the acquisition of Gfp-tagged *Asaia*. To give an example of the colonization pathway, insects submitted to a 48 hours co-feeding were employed for this analysis. Hybridization experiments on midgut and gonad tissue showed the constant presence of *gfp* gene signals together with the natural symbiotic strain (Figure 4A-F). The occurrence of *gfp* gene signals in the digestive tract confirms that the bacterium was ingested during feeding events, and was able to establish in the gut, a favourable environment for acetic acid bacteria [2]. Furthermore, the detection of the *gfp* gene hybridization signal in the gonads revealed that *Asaia*, by passing through the hemocoel, is able to reach the reproductive system from which can be further distributed by both venereal and vertical transmission. Indeed, the occurrence of *gfp* gene signals on the epithelium of testis ducts indicates a possible transfer to females during mating, while the presence in ovaries suggests a vertical transmission *via* egg-smearing, as previously indicated [2,4]. On the other hand, we were not able to detect a positive signal after hybridization with the *gfp* gene-specific probes in salivary glands of insects exposed to co-feeding trials. These results may reflect that *Asaia* needs a longer incubation period to reach salivary glands and to allow onward transmission *via* co-feeding.

Control experiments were performed on 112 leafhoppers sharing sterile sugar solutions (Table 3). Neither the insects nor the corresponding diet samples showed *gfp* positive signals by q-PCR. FISH experiments performed on these individuals confirmed the absence of fluorescent signals after hybridization with the *gfp*-specific probes, while the presence of bacteria, and in particular of the wild type strain of *Asaia*, naturally associated with the insect, was detected in the same insect specimens (Figure 4).

**Table 3:** Experimental design of S. titanus transmission trials.

No. of individuals (donors + receivers)	Transmission type	Acquisition time	Destination
20 (10 + 10)	Co-feeding with *Asaia*	24 hours	q-PCR
38 (19 + 19)		48 hours	
28 (14 + 14)		72 hours	
20 (10 + 10)		96 hours	
8 (4 + 4)		48 hours	FISH
Tot. co-feeders: 114 (57 + 57)			

10 (5 + 5)	*Asaia* Venereal transfer (male to female)	24 hours	q-PCR
10 (5 + 5)		48 hours	
10 (5 + 5)		72 hours	
14 (7 + 7)		96 hours	
10 (5 + 5)		48 hours	FISH

10 (5 + 5)	*Asaia* Venereal transfer (female to male)	24 hours	q-PCR
14 (7 + 7)		48 hours	
10 (5 + 5)		72 hours	
12 (6 + 6)		96 hours	
8 (4 + 4)		48 hours	FISH
Tot. mated: 108 (54 + 54)			

6 (3 + 3)	Co-housing control trial (males with males)	24 hours	
6 (3 + 3)		48 hours	
6 (3 + 3)		72 hours	
6 (3 + 3)		96 hours	

10 (5 + 5)	Co-housing control trial (females with females)	24 hours	q-PCR
6 (3 + 3)		48 hours	
6 (3 + 3)		72 hours	
6 (3 + 3)		96 hours	
Tot. co-housed: 52 (26 + 26)			

20 (10 + 10)	Negative control for Co-feeding	24 hours	q-PCR
22 (11 + 11)		48 hours	
28 (14 + 14)		72 hours	
32 (16 + 16)		96 hours	
10 (5 + 5)		48 hours	FISH
Tot. co-feeders: 112 (56 + 56)			

16 (8 + 8)	Negative control for *v*enereal transfer (male to female)	24 hours	q-PCR
10 (5 + 5)		48 hours	
8 (4 + 4)		72 hours	
14 (7 + 7)		96 hours	
10 (5 + 5)		48 hours	FISH

8 (4 + 4)	Negative control for venereal transfer (female to male)	24 hours	q-PCR
14 (7 + 7)		48 hours	
12 (6 + 6)		72 hours	
10 (5 + 5)		96 hours	
10 (5 + 5)		48 hours	FISH
Tot. mated: 112 (56 + 56)			

Number of insect specimens used for each trial. The duration of the acquisition period, as well as the type of analysis carried out, are indicated both for samples submitted to experiments performed with Gfp-tagged *Asaia* and for negative controls.

### Venereal transmission trials

When Gfp*-*tagged *Asaia*-infected males were mated with uninfected females, transfer of Gfp-tagged symbiotic cells was observed, although a longer period was required to reach infection rates similar to those of the co-feeding trials. After a 24 hour incubation time subsequent to mating, only 20% of females (1 out of 5 individuals) were *gfp* gene-positive, with 40% (2 out of 5) positive after 48 hour, 60% (3 out of 5 individuals) at 72 hours, with 4 out of 7 individuals infected at 96 hours (Figure 1B). The average concentration of the marked symbiont in the body of *S. titanus* also increased with longer incubation periods, even though it remained significantly lower than that of donor individuals (df= 18; F= 11.663; P<0.05) (Figure 1E). In fact, after a 96 hour-incubation time the average titre of Gfp-labelled *Asaia* was of 9.0 × 10^2 ^*gfp* gene copies per pg of insect 18S rRNA gene (Table 1). The ratio between the Gfp strain and total *Asaia* aslo underwent a regular increase, as it passed from a very low value after 24 hours to a percentage higher than that of donor males (17% after 96 hours) (Figure 2B). The average ABR was lower (Table 2) than that reported previously [4], and the average GfpABR was a little lower than the ratio of co-feeders (Table 2). Nonetheless, even though the concentration of the Gfp-tagged *Asaia* did not significantly increase, a slow increment was observed, suggesting a bacterial growth within the host after venereal transfer, which indicates that venereal infection from male to female may be followed by stable colonization. Moreover FISH experiments suggest that Gfp-tagged *Asaia* transmission in female individuals mated with infected males starts from the colonization of gonads, where a massive fluorescent signal after hybridization with the *gfp* gene-specific probe was observed (Figure 4 G-I). FISH results on gonads are in agreement with the actual occurrence of a venereal transfer, however to avoid misinterpretation of data, and to rule out the possibility that the transmission have took place by co-feeding when the two insects were caged in the same capsule, co-housing control trials were set up, both with pairs of male and female individuals. As co-housing specimens were of the same sex, at the end of the trial we were not able to discriminate between donor and recipient individuals, so all were submitted to qPCR for the *gfp* gene. For each pair of individuals, one was always *gfp*-positive (the donor) and the other was *gfp*-negative (the recipient) (Fig 1A). The *gfp* concentration data relative to donor individuals are included in the “donors” raw in Table 1. This result indicates that when the individuals were caged together but cannot mate, transmission did not occur. In effect, in the capsule environment, the copulation between individuals of the opposite sex is more likely than the co-feeding in the same grape leaf: two individuals may never be in contact with the same leaf portion during the relatively short period when they are caged together, on the other hand the capsule is small enough to make the mating very likely.

The results concerning the diets used in venereal transmission experiments from infected males to females showed that no positive signals were detected in samples corresponding to 24 or 48 hours of incubation by quantitative PCR. A possible explanation could be that the bacterial colonization takes longer periods when it starts from the gonads (rather than the gut), passing through the hemocoel and finally reaching the salivary glands. Only when the salivary glands are colonized is the symbiont released into the feeding medium. After 72 hours, one of the five diets was *gfp* gene-positive (20%), and after 96 hours the infection rate raised a value of 29% (2 out of 7) (Figure 1B). The low release rate of Gfp-tagged *Asaia* in the diets was consistent with data from FISH experiments which did not show any signal with the *gfp* gene-specific probes in the salivary glands of the tested females (data not shown). In constrast, in positive diets Gfp-tagged *Asaia* cells reached a concentration of 7.3 × 10^2 ^*gfp* gene copies per ng of DNA sample 96 hours after acquisition (Table 1). Moreover, the density values obtained after a 72-hour feeding were not significantly different from those observed after 96 hours and after co-feeding (df= 42; F= 0.784; P= 0.463) (Figure 1E). The percentage of Gfp-tagged *Asaia* respect to the total population of this symbiont, was very low after 72 hours of incubation (0.2%), became noteworthy after 96 hours, reaching values similar to those obtained after a co-feeding transmission (29%) (Figure 2B). This abundance suggests that oral and venereal routes can act together to horizontally transmit the symbiont. Nevertheless, the percentage of Gfp-labelled and wild type *Asaia* within the bacterial community of diet samples was lower than the values obtained in co-feeding experiments (Table 2). This may be due to fact that the duration of venereal transfer tests was too short to reach similar conditions.

To investigate if Gfp-labelled *Asaia*-infected females can infect males during mating, a reciprocal transfer experiments was carried out. In this case, an irregular infection pattern was observed. Only after 48 and 96 hours of incubation following mating experiments were positive males observed (4 out of 7 *gfp* gene-positive individuals after 48 hours; 3 out of 6 *gfp* gene-positive specimens after 96 hours), while no transmission was detected after 24 and 72 hours (Figure 1C). Such a scattered distribution of colonized males suggests a lower transfer of the Gfp-tagged strain, or could be related to the low number of analysed samples. Furthermore, the titre of Gfp-tagged *Asaia* cells within the body of infected insects decreased by one order magnitude from 48 to 96 hours (Table 1), and in both cases it was significantly lower than that of donor individuals (df= 16; F= 9.947; P<0.05) (Figure 1F). This seems to indicate at least a partial failure of the introduced strain to establish within the host; nevertheless, this possibility is in contrast to the increase of the Gfp to total *Asaia* ratio, which is higher after a 96 hour-incubation (23%) than after 48 hours (0.2%), and with the average GfpABR, which is higher than in the venereal transfer trials from male to female (Table 2). More likely, the unstable trend of data that we obtained is related to a random distribution and can not be considered as a trend, even though copulation must have a role in the bacterial transfer, since co-housing experiments made with pairs of male insects did not show the occurrence of transmission. However, correspondence between infected males and *gfp* gene-positive diets was detected, showing that, whatever the source of infection for these individuals, *Asaia* is able to spread in the insect body and to reach salivary glands to be then injected into the diets. In fact, 1 out of 7 diets was *gfp* gene-positive after a 48 hour-incubation (14.7 *gfp* gene copies per ng of DNA sample), and 2 out of 6 samples after 96 hours (4.1 × 10^2 ^*gfp* gene copies per ng of DNA sample) (Figure 1C, Table 1). No significant difference was observed between the observed concentrations of the Gfp strain (df= 42; F= 0.784; P= 0.463) (Figure 1F). The percentage of Gfp-tagged strain in total *Asaia* was 4% after a 48 hour-incubation, and 32% after 96 hours (Figure 2C), while the GfpABR and the ABR percentages were 0.49 and 3% respectively (Table 2). The uneven and probably random distribution of effective venereal transmission events from infected females to uninfected males was also reflected in the absence of hybridization signal obtained with the *gfp* gene-specific probes when FISH experiments were carried out on male individuals mated with females colonized by Gfp-tagged *Asaia*.

Control experiments were performed by mating 56 insects with the same number of specimens of the opposite sex previously fed on sterile sugar solutions (Table 3). No *gfp*-positive samples were observed when analysing those insects and their respective diets by q-PCR, nor fluorescent signals was detected after hybridization with the *gfp*-specific probes on these samples (Figure 3 D-G).

## Conclusions

Horizontal transmission of *Asaia* occurs in populations of the leafhopper *S. titanus*, as previously reported for mosquitoes [6,20]. Co-feeding experiments demonstrated a high incidence of uptake of the Gfp-tagged *Asaia* by individuals that were fed on diets previously exposed to infected donor insects, with a colonization level which almost reached that of the donor insects. *Asaia*-*S. titanus* is one of the few symbiont-host models in which a direct demonstration of horizontal transmission is provided. In general the horizontal transmission is, in fact, indirectly deduced by analysing the distribution of a symbiont among host taxa and the level of phylogenetic congruency between the insect hosts and the bacterial symbiont [9].

Beside the *Asaia* spread *via* co-feeding, the results of the present study indicate venereal transmission in *S. titanus*, like in the dipteran mosquitoes [20]. Infection can transfer from infected male to female during mating, even if venereally infected individuals do not attain the concentration of acquired bacteria observed following co-feeding. Moreover, venereal transfer may lead to the coexistence of horizontal and vertical transmission. However, the capability of *Asaia* to be acquired by offspring after a venereal transfer from infected males to females was not evidenced in this study, due to difficulties connected with rearing *S. titanus* in laboratory conditions, and thus it can be only presumed. On the other hand, efficient venereal transmission from female to male cannot be ruled out: the uneven distribution observed in the venereal transmission could be due to the low number of individuals employed in the experiments.

These results on the transmission routes of *Asaia* in *S. titanus* encourage research towards the understanding of the ecology of the symbiont in its insect host. Further experiments are needed to evaluate the role(s) of the bacterial symbiont in the insect and how it can affect the host fitness*.*

## Methods

### Construction of the chromosomal Gfp-tagged Asaia strain

*Asaia* strain SF2.1(cGfp) was generated with the purpose of having a stably labeled bacterium by a site-specific tagging through the use of a mini-Tn7 transposition system, as described by Lambertsen *et al. *[26]. Experiments of bacterial competitiveness and stability determined that *Asaia* SF2.1(cGfp) and *Asaia* wild type strain showed comparable growth rate and fitness. The stability of the transformed strain, *Asaia* SF2.1(cGfp), was determined in GLY medium (25 g·liter^-1^ glycerol, 10 g·liter^-1^ yeast extract, pH 5) as reported by Crotti *et al. *[4]. The bacterial competitiveness of *Asaia* SF2.1(cGfp) was evaluated in GLY medium as indicated by Lambertsen *et al. *[26].

### Insect material and transmission trials

Nymphs of *S. titanus* were collected in early summer from vineyards in the Piedmont region between 2009 and 2010, and reared on healthy grape plants in laboratory cages at the DIVAPRA in growth chambers at 25°C and a photoperiod of 16:8 (L:D) h until adult emergence.

The transmission trials carried out with the newly-emerged adults were performed by using *Asaia* strain SF2.1(cGfp). Emerged insects were used as donor individuals and maintained for 48 hours on a sugar diet added of Gfp-tagged *Asaia* as described by Crotti *et al*. [4]. After the 2-day acquisition of the marked symbiont, donor individuals were destined to co-feeding or venereal transmission experiments, as shown in Table 3.

One hundred and fourteen individuals were dedicated to co-feeding trials. They were collected and submitted for further 48 hours to new sterile sugar diets under the selection of kanamycin (100 mg ml^-1^) in order to permit the release in the medium of bacterial cells residing in the salivary glands. After the bacterial release in the diet, donors were collected and preserved as indicated below. At the same time, diets were supplied to new uninfected individuals. These recipient were maintained on these diets for different periods (24, 48, 72, or 96 hours). At the end of these periods, specimens were taken and preserved for the following investigations, partly *in toto* at -20°C for q-PCR analyses, and partly as dissected organs for FISH experiments. The sugar solutions used to feed these insects were taken as well and conserved at -20°C until following analyses.

One hundred and eight donor insects were used in venereal transmission trials and were isolated for 2 days in suitable Petri dishes together with an uninfected individual of the opposite sex to allow mating. Both crosses of males infected with Gfp-tagged *Asaia* with non infected females and mating between colonized females and non infected males were set up. For food supply, capsules were provided with grapevine leaves whose petiole was placed inside an Eppendorf tube containing a nutritive solution [27] and sealed with parafilm to maintain leaf turgor during the experiments. At the end of the mating period, individuals mated with infected *S. titanus* were fed on sterile sugar diets for different periods (24 to 96 hours), in order to permit the insect's body colonization by the bacteria acquired during mating. After the incubation periods, both insects and diets were collected and conserved as described above. To control whether the Gfp *Asaia* transfer really took place by mating, rather than by co-feeding while the two individuals remained in the same capsule, co-housing trials were set up. Further 12 males and 14 females, after the acquisition of the Gfp-marked bacterium, were placed in Petri dishes together with an uninfected individual of the same sex, under the same conditions of the venereal transfer experiments. After 2 days (without copulation), both the specimens were fed on sterile sugar diets for different periods (24 to 96 hours), like for the other trials.

For each co-feeding experiment, other 56 individuals fed on sterile sugar diets were used as donors in trials designed as negative control; similarly, for each venereal transmission experiment, 56 individuals fed on sterile solution were mated with specimens of the opposite sex as negative control (Table 3). After mating of negative control individuals, receiving specimens were maintained singularly on sugar diets for periods varying from 24 to 96 hours to simulate the transmission trials.

### Quantitative real-time PCR for the Gfp-tagged Asaia

Subsequent to the transmission trials, *S. titanus* individuals and sugar diets for molecular analyses were submitted to total DNA isolation. Nucleic acids extraction was performed by sodium dodecyl sulfate-proteinase K-cethyltrimethyl ammonium bromide treatment [28], which for insects was modified as described in Raddadi *et al*. [29]. The precipitated DNA was resuspended in 50 µl (insect samples) or in 20 µl (diet samples) of TE buffer, pH 8 and kept at -20°C until use.

Quantitative real-time PCR was performed on a Chromo4 real-time detector (Bio-Rad, Milan, Italy) to measure the presence and concentration of Gfp-tagged *Asaia* in insects and diets. The reactions were performed with IQ^TM^ SYBR® Green Supermix (Bio-Rad), using primers targeting the *gfp* cassette (GFP540F / GFP875R) [30] and the insect’s 18S rRNA gene (MqFw / MqRv) [31]. The latter were used to normalize the *gfp* concentration values for the total DNA amount of each sample. To calculate the relative abundance of Gfp-labelled *Asaia* respect to the total *Asaia* cells and the whole bacterial community, *Asaia*-specific and eubacterial primers were used also, according to Favia *et al. *[6]. To construct standard curves, the *gfp* gene of *Asaia* strain SF2.1(cGfp) and the 16S rRNA gene of the wild type bacterium amplified by PCR were cloned using the pGEM T-easy Vector Cloning Kit (Promega). After the determination of *gfp* and 16S rRNA gene copies of Gfp*-*tagged *Asaia*, total *Asaia*, and bacteria, the following ratios were calculated: Gfp-labelled *Asaia* to total *Asaia* ratio, Gfp-labelled *Asaia* to bacteria ratio (GfpABR), and *Asaia* to bacteria 16S rRNA gene copy ratio (ABR), the latter according to Favia *et al*. [6]. These ratios were used to estimate the relative abundances of the introduced strain within total *Asaia* population in *S. titanus* individuals and of Gfp-labelled *Asaia* and *Asaia* sp. in the bacterial community associated with the insect samples.

### Statistical analyses

To compare the Gfp *Asaia* density detected in co-feeding or venereal transmission experiments for every tested period, q-PCR data relative to the gfp gene concentration were log-transformed, after adding the constant 10, and analyzed by one-way analysis of variance (ANOVA). In addition, means were separated by Tukey test (P<0.05) when variance homogeneity was satisfied (Levene test, P<0.05).

### Fluorescent in situ hybridization

Fluorescent *in situ* hybridization analysis was carried out on organs dissected in a sterile saline solution from donor and recipient *S. titanus* individuals that were not used for Real time PCR experiments. The dissected organs were fixed for 2 min at 4°C in 4% paraformaldehyde and washed in PBS. All hybridization experiment steps were performed as previously described [4] using specific and universal fluorescent probes. For detection of Gfp-labelled *Asaia*, probes *gfp*540 (5’-CCTTCGGGCATGGCACTCTT-3’) and *gfp*875 (5’-GGTAAAAGGACAGGGCCATCGCC-3’) were labelled with Cy5.5 (indodicarbocyanine, absorption/emission at 675-694 nm). Probes Asaia1 and Asaia2, labelled with Cy3 (indocarbocyanine, absorption/emission at 550/570 nm), were used to observe the total *Asaia* population hosted by *S. titanus* individuals [6]. As a positive control for the hybridization experiment, a universal bacterial probe EUB388 labelled with fluorescein isothiocyanate (FITC, absorption/emission at 494/520 nm) was also used [32]. After hybridization, the samples were mounted in antifading medium and then observed in a laser scanning confocal microscope SP2- AOBS (Leica).

## Competing interests

The authors declare that they have no competing interests.

## Authors’ contributions

EG designed and performed most of the experiments, analyzed data and wrote the manuscript. EC and AR provided the *Asaia* strain SF2.1(cGfp) and designed the experiments, MM designed FISH experiments and performed confocal microscopy observations. GF gave suggestions and contributed to data analysis. AA and DD designed and supervised all the experiments. All authors have read and approved the final manuscript.
